# Digit Sucking Habit and Association with Dental Caries and Oral Hygiene Status of Children Aged 6 Months to 12 Years Resident in Semi-Urban Nigeria

**DOI:** 10.1371/journal.pone.0148322

**Published:** 2016-02-18

**Authors:** Kikelomo Adebanke Kolawole, Morenike Oluwatoyin Folayan, Hakeem Olatunde Agbaje, Titus Ayodeji Oyedele, Elizabeth Obhioneh Oziegbe, Nneka Kate Onyejaka, Nneka Maureen Chukwumah, Olusegun Victor Oshomoji

**Affiliations:** 1 Department of Child Dental Health, Faculty of Dentistry, Obafemi Awolowo University, Ile-Ife, Osun State, Nigeria; 2 Department of Child Dental Health, Obafemi Awolowo University Teaching Hospitals Complex, Ile-Ife, Osun State, Nigeria; Navodaya Dental College and Hospital, Mantralayam Road, INDIA

## Abstract

**Objectives:**

Non-nutritive sucking (NNS) is a common behavior in childhood. The association between digit sucking, dental caries and oral health has been studied with inconclusive results. The objectives of this study were to determine the prevalence of, and the association between digit sucking, caries and oral hygiene status of children age six months to 12 years, resident in Ile-Ife, Osun State, Nigeria.

**Methods:**

A cross-sectional study was conducted in Ife Central Local Government Area of Osun State. Data were collected through a household survey using a multi-stage sampling procedure from children between six months and 12 years. Details of each child’s socio-demographic characteristics, digit sucking habits, caries status and oral health status were collected. The association between digit sucking, caries status and oral hygiene status was determined using Chi square and Logistic regression.

**Results:**

The mean age of the 992 study participants was 5.8 ± (3.2) years. The prevalence of digit sucking, caries and poor oral hygiene were 7.2%, 10.5% and 2.4% respectively. The mean dmft score was 0.22 ± (0.80), mean DMFT score was 0.04 ± (0.30) while mean Oral Hygiene Index score was 1.27 ± (0.73). Digit sucking increased the odds of having caries (OR: 1.28; CI: 0.58–2.81) but decreased the odds of having poor oral hygiene (OR: 0.58; CI: 0.34–1.01) insignificantly.

**Conclusions:**

Digit sucking was not a significant predictor of caries and oral hygiene status, although the odds of having caries increased while the odds of having poor oral hygiene decreased with digit sucking.

## Introduction

A habit is an inclination or aptitude for some action acquired by frequent repetition, showing itself in increased facility to performance and reduced power of resistance [1]. One of the commonest oral habits is sucking, a reflex present at birth, although oral contractions and other sucking reflexes have been observed before birth [2]. Sucking habits could be nutritive (breast and bottle feeding) or non-nutritive.

The commonest form of non-nutritive sucking (NNS) is digit sucking [3, 4]. Several studies have evaluated its etiological factors and suggest that fatigue, boredom, excitement, hunger, fear, physical and emotional stress, and insufficient satisfaction of sucking need in infancy are situations that could stimulate digit sucking habits. Sucking may provide happiness and a sense of security when a child faces difficult times [5, 6]. It may also give a feeling of warmth and contentment [7].

Detrimental effects of digit sucking include disturbances in arch form, recurrent otitis media, the possibility of accidents, development of latex allergy, tooth decay, oral ulcers and sleep disorders [8]. Others include wrinkled, chapped or blistered fingers, ulceration, corn formation, dishpan thumb as well as reduced peer acceptance [2, 9]. Digit sucking may also accompany behaviors like trichotillomania [10].

The association between digit sucking and dental caries has been studied but results have been inconclusive. Yonezu and Yakushiji [11] found that children with finger-sucking habits were more likely to be free of caries by age three years. Their finding was associated with increased inter-dental spacing which resulted from flaring of teeth due to digit sucking. On the contrary, a study conducted in Baghdad reported an increase in caries severity with NNS [4]. NNS was associated with malocclusion, making tooth cleaning difficult and allowing accumulation of dental plaque.

The aims of this study were to determine the prevalence of digit sucking, caries and oral hygiene status in the study population, and the association between digit sucking, caries and oral hygiene status of children from six months to 12 years, resident in Ife Central Local Government Area (LGA) of Osun State, Nigeria.

## Methodology

### Study Design

This was a cross sectional study that recruited participants from National Population Enumeration sites in Ife Central LGA. These geographical sites were selected because the participants there were familiar with the conduct of such surveys.

### Study Setting

Ife Central LGA is a semi-urban area of Osun State, which hosts the Obafemi Awolowo University and its Teaching Hospital. The 1991 census put the population of the LGA at 96,580 while the estimated population for the year 2004 was 138,818. The child population for the LGA is about 14,000.

### Study Population

Participants were included in the study if they were between the ages of six months and 12 years, living with their biological parents or legal guardians who consented to participate in the study.

### Sample Size Determination

The sample size was calculated using Leslie Fischer's formula[12] for study population >10,000. Based on a prevalence of 34.1% of oral habits of children aged four to 15 years old, determined by Quashie-Williams *et al* [3], a sample size of 1,011 children was necessary to identify 345 children with oral habits, giving a non-response rate of about 10%.

### Sampling Technique

The sampling procedure was a (three-level) multi-stage cluster sampling aimed at selecting eligible persons with known probability. Stage 1 involved the random selection of enumeration areas within the LGA.At the sites, every third household on each street was selected. Stage 2 involved listing eligible individuals within households. Stage 3 involved selection of actual respondents for interview. Only children present in the house at the time of study were eligible and one child per household was selected. Details of this sampling technique has been reported by Folayan *et al*. [13] in an earlier publication from this same database.

### Data Collection

Data were collected through face to face interviews using a structured questionnaire. Experienced field workers who had been engaged in past national surveys were recruited and trained on the study protocol. The interviewers collected all information from respondents and submitted to survey supervisors who reviewed the questionnaires. Mothers were requested to respond on behalf of children below eight years, based on evidence that responses of mothers on questionnaires have a higher correlation with children’s responses [14]. However, where the mother was unavailable, fathers completed the questionnaires. Each child’s socio-demographic characteristics were obtained.

### Socio-economic Status

Socio-economic status was determined using an adapted version of a socio-economic index described by Olusanya *et al*.[15]. The tool has been tested and found valid and reliable in Nigeria [16, 17]. Data were collected on educational level and profession of respondent’s parents. The mother’s level of education was classified as ‘no formal education, Quranic or Primary school education’ and scored as 2; Secondary school education was scored as 1; and Tertiary education scored as 0. Father’s occupation was categorized into three: Civil servants or skilled professionals with tertiary level of education scored as 1; Civil servants or skilled professional with secondary level of education scored as 2; Unskilled, unemployed individuals, students, and civil servants or skilled professional with primary or Quaranic education were scored as 3. The scores for the mother and father were summed to give social classes I–V, where classes I and II represented the upper class, class III the middle class and IV and V, the lower socio-economic class. When a child had lost a parent, the socio-economic status was determined from the living parent.

### Digit Sucking Habit

Questions were asked about type and number of digits sucked and the frequency of engaging in the habit. Options were, ‘irregularly’, ‘once a week’, ‘2–3 times a week’, ‘once a day’ and ‘several times a day’. Duration of sucking was explored and options ranged from ‘less than a minute’ to ‘1–5 minutes’, ‘5–10 minutes’, ‘10–20 minutes’, ‘20–30 minutes’, and ‘almost continuously’. The intensity of the sucking habit was explored by asking if or not an audible sucking or popping sound was heard while sucking.

### Dietary History

Dietary history was obtained with a dietary chart which recorded all study participants meals and snacks, taken both at meal times and in between meals for three consecutive days, including two week days and a weekend or public holiday.

### Intra-oral Examination

Intra-oral examination was conducted in the homes of all participants to determine the presence of caries and oral hygiene status. The participants were examined sitting, under natural light, using sterile dental mirrors and probes by trained dentists. The teeth were examined wet and debris removed with gauze where present. Radiographs were not used in this study.

### Caries Profile

Caries diagnosis was based on the recommendation of the WHO Oral Health Survey methods [18]. The decayed, missing and filled teeth index (dmft/DMFT) was used. The number of decayed, missing or filled teeth were summed together to give the dmft/ DMFT score for the primary/permanent dentition. For the purpose of analysis, caries status was further divided into caries present or absent.

### Oral Hygiene Status

The Oral Hygiene Index–Simplified (OHI-S) described by Greene and Vermillion [19] was used to determine the oral hygiene status. Its components, the Debris Index and Calculus Index, were obtained based on six numerical determinations representing the amount of debris or calculus found on the surfaces of index teeth 11, 16, 26, 31, 36, 46 and 51, 55, 65, 71, 75, 85 in the permanent and deciduous dentitions respectively. Debris and calculus scores were totaled and divided by the number of surfaces scored. Scores were graded as 0.0–1.2 = Good oral hygiene, 1.3–3.0 = Fair oral hygiene and > 3.1 = Poor oral hygiene.

### Standardization of Examiners

Clinical investigators were qualified dentists undergoing postgraduate residency training as Paedodontists or Orthodontists who were calibrated on the study protocol and the WHO criteria for caries diagnosis, including the use of the dmft/DMFT index and OHI-S. Training was followed by practice on patients. Each investigator examined and scored children for oral lesions as prescribed in the study protocol. Results were subjected to a Cohen’s weighted kappa score analysis to determine intra- and inter- examiner variability. The intra-examiner variability ranged between 0.89–0.94, while inter-examiner variability ranged between 0.82–0.90 for caries detection and OHI-S.

### Theoretical Model for Statistical Analysis

**Fig 1:**
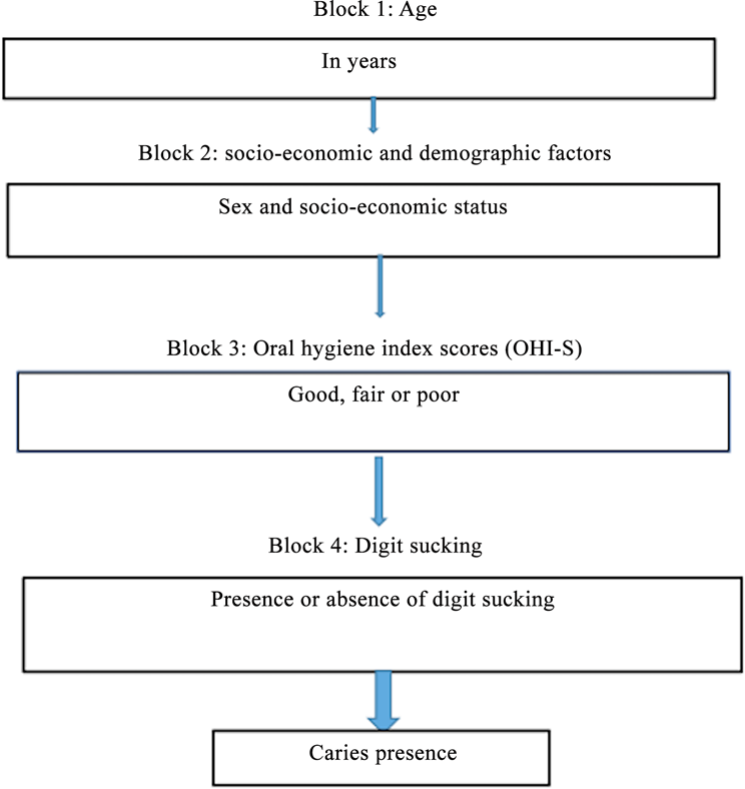
Hierarchical model for analysis of predictors of caries presence.

A hierarchical theoretical model with the following four blocks was employed for the analysis of predictors for the presence of dental caries: 1) age of child. 2) socio-economic and demographic factors, 3) Oral Hygiene Index and 4) oral habit (Fig 1). Age was considered a potentially confounding factor [20, 21], which informed the adjustment of the developed model for this variable. The second block included socio-economic and sex as distal factors in the theoretical model since they could influence oral hygiene status [22] and the digit sucking habits [23, 24]. Oral hygiene practice may be a moderator variable of the association between digit sucking and caries [4], so it was included in the third block. In the fourth block, the digit sucking was recorded as the oral habit with the assumption that this variable may influence caries risk.

**Fig 2:**
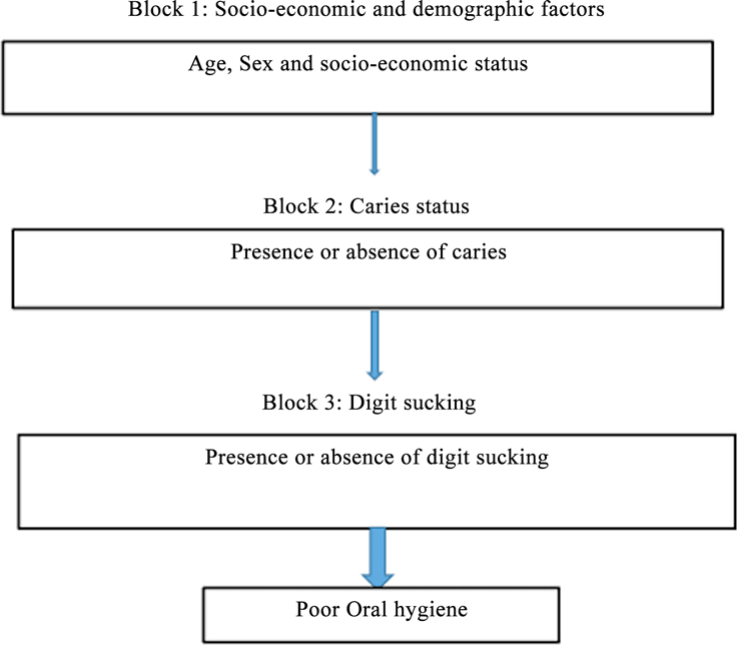
Hierarchical model for analysis of predictors of poor oral hygiene.

A hierarchical theoretical model with the following three blocks was also employed for the analysis of predictors of poor oral hygiene: 1) Socio-economic and demographic factors, 2) caries status and 3) oral habit. The fundamentals that informed the developed model for predicting poor oral hygiene were the same for predicting the presence of caries. Socio-economic status, age and sex are potentially confounding factors for caries [21, 25, 26] and oral hygiene status [22]. In the second block, caries status was included as a moderator variable for oral hygiene [27]. In the third block, digit sucking was recorded as the investigated oral habit that may influence the risk for poor oral hygiene. (Fig 2).

### Data Analysis

The ages of the 10 children six to 11months were rounded off to 1 year for ease of data analysis. Descriptive analyses were conducted to determine the prevalence of digit sucking, caries and oral hygiene status. Bivariate analysis was conducted to test the association between dependent variables (presence of caries and oral hygiene status) and the independent variables (child’s age, gender, socio-economic status). Where appropriate, chi square tests were conducted.

Logistic regression was used for inferential analysis. The hierarchical modeling started with the first block whose variables were adjusted simultaneously for each other. Only those variables whose p value were <0.4 entered into the subsequent models. Variables of the second block were adjusted simultaneously for each other and for the variables whose p value were <0.4 in the previous step. Digit sucking was included in both models irrespective of the p value in the previous steps because of the need to evaluate the effect of digit sucking on the presence of caries and poor oral hygiene. The significance of each variable was considered at the time of entry in the model (p value ≤0.05). All other blocks were then added in succession, following the same procedure. The estimated coefficients were expressed as odds ratios (ORs) and their 95% confidence intervals were also calculated. The Hosmer-Lemeshow goodness-of-fit test was done to confirm the consistency of the models. Where data were skewed, the dichotomized version was used. Statistical analysis was conducted with SPSS (version 17.0) for windows, while STATA software (version 10) was used for the logistic regression. Statistical significance was inferred at p ≤ 0.05.

### Ethical Consideration

Ethical approval was obtained from the Ethics and Research Committee of the Obafemi Awolowo University Teaching Hospitals Complex Ile-Ife (ERC/2013/07/14). Approval to conduct the study was also obtained from the Local Government Authority. The study was conducted in full compliance with the study protocol. Written informed consent was obtained from the parents of study participantsafter duly explaining study objectives, risks and benefits, voluntary nature of participation and freedom to withdraw at any time. All children aged eight to 12 years also provided written assent. Efforts were made to minimize risks such as loss of confidentiality and discomfort, to participants. All data were collected without the identifier (names and addresses) of participants. Participants experienced no direct benefit and no compensation was paid. However they were given token gifts of stationery or a small tube of toothpaste containing 1450ppm of fluoride. The fluoride level of drinking water sources in most LGAs in the South West geopolitical zone of Nigeria where Ile-Ife is situated is between 0.00pm—0.30ppm [28] highlighting the need to promote further use of topical fluoride. None of the gifts exceeded a value of $0.50.

## Results

Only the data of 992 children recruited for the study were complete enough for analysis. This represents 90.1% of the proposed 1,011 study participants. None of the children recruited refused to participate. Participants included 508 (51.2%) boys and 484 girls (48.2%) with a mean age of 5.83 ± (3.15) years. There were 497 (50.1%) study participants in the 1 to 5 year age group and 495 (49.9%) in the 6 to 12 year age group, with mean ages of 3.15 ± (1.35) and 8.53 ± (1.90) years respectively.

### Digit Sucking Habit and Caries Profile Of Study Participants

Table 1 shows the socio-demographic and digit sucking profile of study participants. Seventy one (7.2%) of them had digit sucking habits. Fifty five (77.5%) engaged in thumb sucking habits while 16 (22.5%) sucked other digits. The majority of children with digit sucking habits (56.3%) fell into the 1 to 5 year age group. The prevalence of digit sucking was highest among the 2 year olds and least among the 7 and 12 year olds. There were no significant differences in the proportion of children aged 1 to 5 years and 6 to 12 years (p = 0.27), male and female participants (p = 0.37) and children in the different socio-economic classes (p = 0.40) who sucked their digits.

**Table 1:** Profile of study participants (N = 992).

Variables	Digit sucking habit present	Digit sucking habit absent	Total	P value
	n = 71 (7.2%)	n = 921 (92.8%)	N = 992 (100%)	
**Age**				
1–5 years	40 (56.3%)	457 (49.5%)	497 (50.1%)	0.27
6–12 years	31 (43.7%)	464 (50.5%)	495 (49.9%)	
**Sex**				
Male	40 (56.3%)	468 (50.8%)	508 (51.2%)	0.37
Female	31 (43.7%)	453 (49.2%)	484 (48.8%)	
Socio-economic status*				
High SES	21 (29.6%)	245 (26.6%)	266 (26.8%)	0.40
Middle SES	30 (42.2%)	389 (42.3%)	419 (42.3%)	
Low SES	20 (28.2%)	286 (31.1%)	306 (30.9%)	
**Caries status**				
Caries present (DMFT>0 and dmft>0)	8 (11.3%)	96 (10.4%)	104 (10.5%)	0.82
Caries absent (DMFT = 0 and dmft = 0)	63 (88.7%)	825 (89.6%)	888 (89.5%)	
Oral Hygiene Index Score**				
Good oral hygiene	47 (67.2%)	494 (54.1%)	541 (55.0%)	0.11
Fair oral hygiene	22 (31.4%)	397 (43.5%)	419 (42.6%)	
Poor oral hygiene	1 (1.4%)	22 (2.4%)	23 (2.4%)	

*SES could only be determined for 991 participants

**OHIS could only be determined for 983 participants

Table 2 highlights the caries profile of participants. One hundred and four children (10.5%) had dental caries. Significantly more females than males (61.5% vs 38.5%; p = 0.01), and more children in the 6 to 12 years than the 1 to 5 years age group had caries (71.2% vs 28.8%; p = 0.00). There was no significant difference in the proportion of children from each of the socio-economic strata who had caries (p = 0.13).

**Table 2:** Caries profile of study participants (N = 992).

Variables	Caries present	Caries absent	Total	P value
	n = 104 (10.5%)	n = 888 (89.5%)	N = 992 (100%)	
**Age**				
1–5 years	30 (28.8%)	467 (52.6%)	497 (50.1%)	<0.001
6–12 years	74 (71.2%)	421 (47.4%)	495 (49.9%)	
**Sex**				
Male	40 (38.5%)	468 (52.7%)	508 (51.2%)	0.01
Female	64 (61.5%)	420 (47.3%)	484 (48.8%)	
Socio-economic status*				
High SES	19 (18.4%)	247 (27.8%)	266 (26.8%)	0.13
Middle SES	48 (46.6%)	371 (41.8%)	419 (42.3%)	
Low SES	36 (35%)	270 (30.4%)	306 (30.9%)	
Oral Hygiene Index Score**				
Good oral hygiene	40 (38.5%)	501 (57.0%)	541 (55.0%)	<0.001
Fair oral hygiene	59 (56.7%)	360 (41%)	419 (42.6%)	
Poor oral hygiene	5 (4.8%)	18 (2.0%)	23 (2.4%)	
**Digit sucking present**				
Yes	8 (7.7%)	63 (7.1%)	71 (7.2%)	0.82
No	96 (92.3%)	825 (92.9%)	921 (92.8%)	

*SES could only be determined for 991 participants

**OHI-S could only be determined for 983 participants

The dmft score ranged from 0 to 8 with a mean score of 0.22 ± (0.80). There were 192 unrestored carious teeth, nine missing and three filled primary teeth. The DMFT score also ranged from 0 to 4 with a mean score of 0.04 ± (0.30). There were 29 unrestored carious teeth, three missing and two filled permanent teeth. Only eight (11.3%) of the 71 children with digit sucking habits had dental caries. There was no significant difference in the proportion of children with or without digit sucking habits who had caries (11.3% vs 10.4% p = 0.82). (Table 1) None of the children with digit sucking habits had missing or filled teeth.

### Digit Sucking Habit and Oral Hygiene Status of Study Participants

The mean OHI-S score was 1.27 ± (0.73). Mean OHI-S score was significantly better in the 1 to 5 years age group compared with the 6 to 12 years age group (0.98 vs 1.56; p < 0.001). No significant gender difference was observed in the mean OHI-S scores (1.28 vs 1.27; p = 0.73). There was a significant difference in mean OHI-S scores (1.07 vs 1.29; p = 0.02) of digit and non-digit suckers. Table 3 highlights the association between oral hygiene status of study participants and dependent variables. The distribution of OHI-S scores was not significantly different across gender (P = 0.89) and socio-economic strata (P = 0.29).

**Table 3:** Oral hygiene profile of study participants (N = 992).

Variables	Good oral hygiene	Fair oral hygiene	Poor oral hygiene	Total	P value
	n = 541 (55.0%)	n = 419(42.6%)	n = 23(2.4%)	N = 983 (100%)	
**Age**					
1–5 years	346 (64.0%)	141 (33.7%)	1 (4.3%)	488 (59.6%)	<0.001
6–12 years	195 (36.0%)	278 (66.3%)	22 (95.7%)	495 (50.4%)	
**Sex**					
Male	273 (50.5%)	218 (52.0%)	12 (52.2%)	503 (51.2%)	0.89
Female	268 (49.5%)	201 (48.0%)	11 (47.8%)	480 (48.8%)	
Socio-economic status*					
High SES	158 (29.2%)	102 (24.4%)	5 (21.7%)	265 (27.0%)	0.29
Middle SES	218 (40.3%)	188 (45.0%)	8 (34.8%)	414 (42.2%)	
Low SES	165 (30.5%)	128 (30.6%)	10 (43.5%)	303 (30.8%)	
**Caries status**					
Caries present (DMFT > 0 and dmft > 0)	40 (7.4%)	59 (14.1%)	5 (21.7%)	104 (10.6%)	<0.001
Caries absent (DMFT = 0 and dmft = 0)	501 (92.6%)	360 (85.9%)	18 (78.3%)	879 (89.4%)	
**Digit sucking present**					
Yes	47 (8.7%)	22 (5.3%)	1 (4.3%)	70 (7.1%)	0.10
No	494 (91.3%)	397 (94.7%)	22 (95.7%)	913 (92.9%)	

*SES could only be determined for 982 participants

### Digit Sucking Habit and Caries Status of Study Participants

The association between the type of digit sucked, habit severity, caries and oral hygiene status was further explored. This revealed that there was no significant association between the type of digit sucked (p = 0.45) or severity of digit sucking (p = 0.53) and caries presence. Neither was there a significant association between the type of digit sucked (p = 0.32) or digit sucking severity (p = 0.79) and oral hygiene status.

### Predictors of Caries

Table 4 highlights the results of logistic regression to determine the predictors of caries. The Hosmer-Lemeshow test confirmed the goodness of fit of the model (p = 0.44). Age, gender and oral hygiene status were the significant predictor of caries for the study population. The odds of having caries increased with increasing age (OR: 1.09; 95% CI: 1.02–1.17; p = 0.01). Females had significantly increased odds of having caries when compared to males (OR: 1.79; 95% CI: 1.17–2.74; p = 0.01). Children with fair oral hygiene status also had significantly increased odds of having caries when compared with children with good oral hygiene (OR: 1.71; 95% CI: 1.09–2.68 p = 0.02).

**Table 4:** Logistic regression determining predictors of caries presence in a sample of 983 children.

Variables	Block 1	*P* value	Block 2	*P* value	Block 3	*P* value	Block 4	*P* value
	OR (95% CI)		OR (95% CI)		OR (95% CI)		OR (95% CI)	
**Age**	1.14 (1.07–1.21)	<0.001	1.13 (1.06–1.21)	<0.001	1.09 (1.02–1.17)	0.01	1.09 (1.02–1.17)	0.01
**Sex**								
Male			-	-	-	-	-	-
Female			1.73 (1.13–2.64)	0.01	1.78 (1.16–2.73)	0.01	1.79 (1.17–2.74)	0.01
**Socio-economic status**								
High			-	-	-	-	-	-
Middle			1.72 (0.98–3.02)	0.06	1.68 (0.96–2.95)	0.07	1.68 (0.95–2.94)	0.07
Low			1.58 (0.88–2.85)	0.13	1.57 (0.87–2.84)	0.13	1.58 (0.87–2.85)	0.13
**Oral hygiene status**								
Good					-	-	-	-
Fair					1.70 (1.09–2.65)	0.02	1.71 (1.09–2.68)	0.02
Poor					2.49 (0.84–7.37)	0.10	2.51 (0.85–7.44)	0.10
**Digit sucking habit**								
Absent							-	-
Present							1.28 (0.58–2.81)	0.54

The odds of having caries also increased for children with middle (OR: 1.68; 95% CI: 0.95–2.94) and low socio-economic status (OR: 1.58; 95% CI: 0.87–2.85) when compared with those with high socio-economic status. Children with poor oral hygiene (OR: 2.51; 95% CI: 0.85–7.44) compared with those with good oral hygiene, and children with digit sucking habits (OR: 1.28; 95% CI: 0.58–2.81) compared with those without digit sucking habits also had increased odds of having caries. However, these findings did not reach statistical significance.

### Predictors of Poor Oral Hygiene

Table 5 highlights the results of the logistic regression to determine the predictors of poor oral hygiene. The Hosmer-Lemeshow goodness-of-fit test confirmed the consistency of fit of the model (p = 0.24). Age and presence of caries were the only significant predictors of poor oral hygiene. Children aged 1 to 5 years had reduced odds of having poor oral hygiene when compared with children aged 6 to 12 years (OR: 0.27; 95% CI: 0.21–0.36; P<0.001)., Children with caries had increased odds of having poor oral hygiene when compared with children without caries (OR: 1.66; 95% CI: 1.07–2.59; P = 0.03). Female children (OR: 0.85; 95% CI: 0.65–1.11) and children who were digit suckers (OR: 0.58; 95% CI: 0.34–1.01) had decreased odds of having poor oral hygiene when compared with male children and non-digit suckers respectively. These findings were however, not significant.

**Table 5:** Logistic regression determining predictors of poor oral hygiene in a sample of 983 children.

Variables	Block 1	*P* value	Block 2	*P* value	Block 3	*P* value
	OR (95% CI)		OR (95% CI)		OR (95% CI)	
Age						
1–5 years	-	-	-	-	-	-
6–12 years	0.26 (0.20–0.34)	<0.001	0.27 (0.21–0.36)	<0.001	0.27 (0.21–0.36)	<0.001
Sex						
Male	-	-	-	-	-	-
Female	0.88 (0.67–1.14)	0.35	0.86 (0.66–1.12)	0.26	0.85 (0.65–1.11)	0.24
Socio-economic status						
High	-	-	-	-	-	-
Middle	1.37 (0.98–1.90)	0.06	1.34 (0.96–1.87)	0.08	1.34 (0.96–1.86	0.09
Low	1.11 (0.78–1.57)	0.57	1.09 (0.76–1.55)	0.64	1.08 (0.76–1.54)	0.67
Caries						
Absent			-	-	-	-
Present			1.65 (1.06–2.56)	0.03	1.66 (1.07–2.59)	0.03
Digit sucking habit						
Absent					-	-
Present					0.58 (0.34–1.01)	0.052

## Discussion

This study is the first to determine the prevalence of digit sucking habit, caries and oral hygiene status of children at a population level in Ile-Ife. The prevalence of digit sucking, caries and poor oral hygiene among the study population was low. Digit sucking was not a predictor of caries and poor oral hygiene status. It however increased the odds of having caries and good oral hygiene in the study population, though these findings were not significant. Being a female and aged six to12 years were significant predictors of the presence of caries for the study population. Having caries and being between six and 12 years old were also significant risk factors for poor oral hygiene.

The use of a household survey for study participants’ recruitment made the findings of the study generalizable to the study environment. This is because the recruitment method increased the probability of including children targeted for the study from all the socio-economic strata in the study population, irrespective of their ability to be enrolled in school or not. The robust analytical approach also reduced the chances of spurious inferences. However, the recruitment of study participants from enumeration sites familiar with research studies inherently introduced a bias into the study sample. Despite this limitation, the study was able to provide very useful information on oral health status and its relationship with digit sucking habits.

One of the highlights of the study is the low prevalence of digit sucking in the study population when compared with prior reports. This supports previous suggestions of variability in the prevalence of NNS habits in different cultures [29–31]. Proffit [32] noted that the prevalence of oral habits is lower amongst less cosmopolitan communities like our study community, where children had ready access to their mothers’ breasts for a long period and rarely suck other objects. A second highlight is the insignificant difference observed in the proportion of children who were digit suckers across the different age groups, gender and socioeconomic strata. This is unlike a prior report of a decline in digit sucking habit with increasing age [24]. More girls than boys were reported to continue digit sucking after beginning school [32]. Adair [23] also reported a higher prevalence of digit sucking habits in children with working class mothers. Children have to compete for their mother’s limited time and may resort to digit sucking for comfort. The low prevalence of digit sucking among the study population may have made it difficult to pick up age, gender and socioeconomic differences in the sub-analysis conducted.

Third, the non-statistical association between digit sucking and caries observed in this study differed from prior findings of Yonezu and Yakushiji [11]. Similar to the reports of Misbah [4], we observed that digit sucking increased the odds of having caries although our results did not reach statistical significance. We however did not observe a significant association between digit sucking and the risk of poor oral hygiene, a probability that Misbah [4] alluded to. We suggest that digit sucking may have been protective due to increased salivary flow resulting from the habit. Future longitudinal studies may however help increase understanding on the association between digit sucking, caries and oral hygiene status.

Fourth, the low caries prevalence in this study is similar to previous reports in many sub-Saharan African countries [33]. We found age and gender to be predictive factors for caries. Age is a known risk factor for caries [34, 35]; as age increases, caries risk increases. The relationship between age and caries risk had been reported in prior studies in our study environment [36, 37]. However, the finding that females have increased risk for caries as shown by this study and other previous studies [26, 38, 39] is still debatable. A few studies had highlighted an increased risk for caries in males [37]. However, increasing evidences seem to show that females are truly at increased risk for caries. Differences in salivary composition, salivary flow rate, hormonal fluctuation, dietary habits and genetic variations increase the risk of females for dental caries [40].

Despite the low caries prevalence, the range of the dmft and DMFT reported in this study implies that there is the need to actively identify children at risk for caries and manage them. Oziegbe and Esan [41] reported higher ranges of dmft/DMFT for children in the same environment as ours. Their study however included children aged 4 to 16 years, which meant that they had more children whose teeth had been exposed to the oral environment for a longer duration, than the children in our study population. Increased duration of tooth exposure to the oral environment implies that teeth face greater risk of acidic assault and caries formation [34, 35]. The mean dmft score of our study population was also higher than the mean DMFT, a finding that has also been observed by others [42].

Fifth, caries presence and being 6 to 12years old were predictive factors for poor oral hygiene. This has been earlier reported by Gopinath *et al* [27]. Worse oral hygiene status in older children may be due to poor oral hygiene practices as children become independent of parental oral hygiene supervision [43, 44]. This underscores the importance of using various motivational methods to promote oral hygiene practices in adolescents. The risk of caries is heightened when the oral hygiene is poor [36, 45].With the outcome of this study showing that caries presence increases the odds of having poor oral hygiene, the prospect of a cycle of caries and poor oral hygiene being set up throughout the life course of children in the study environment, if specific interventions are not instituted in adolescence to improve the oral hygiene, is high.

In conclusion, though the findings of this study have increased public information about the associations between digit sucking, caries and oral hygiene status, it was not able to provide conclusive evidence on the association between the variables. The non-significant association between digit sucking, caries and oral hygiene status, and prior reports, on the detrimental effect of digit sucking on oral health [8] makes it important to promote discontinuation of the habit as soon as feasible.
